# Psoriasis and dementia: A population‐based matched cohort study of adults in England

**DOI:** 10.1002/acn3.52283

**Published:** 2025-01-01

**Authors:** Julian Matthewman, Kathryn E. Mansfield, Sharon L. Cadogan, Katrina Abuabara, Catherine Smith, Krishnan Bhaskaran, Sinéad M. Langan, Charlotte Warren‐Gash

**Affiliations:** ^1^ Department of Non‐Communicable Disease Epidemiology London School of Hygiene and Tropical Medicine Keppel Street London WC1E 7HT UK; ^2^ Department of Dermatology University of California San Francisco 1701 Divisadero Street San Francisco California 94115 USA; ^3^ King's College London St John's Institute of Dermatology 2 Lambeth Palace Rd London SE1 7EP UK

## Abstract

**Objective:**

Evidence for an association between psoriasis and dementia is limited and conflicting. We aimed to investigate the association using large and representative population‐based data and describe risk by dementia subtype and over time.

**Methods:**

We compared dementia risk between people with and without psoriasis using an age‐, sex‐ and primary care practice‐matched cohort of adults aged ≥40 years from the Clinical Practice Research Datalink Aurum in England (1997–2021) linked to hospital admissions data, analysed with stratified Cox regression.

**Results:**

Among 360,014 individuals with psoriasis and 1,799,617 without, psoriasis was associated with a small increased risk of all‐cause dementia (adjusted hazard ratio [aHR] 1.06, 95% CI 1.04–1.08; absolute rate difference 24 per 100,000 person‐years). Strength of association increased with time since psoriasis diagnosis (e.g. aHR 0.99, 0.96–1.03 within 0 to 5 years; 1.20, 1.05–1.37 within 20 to 25 years). The association was stronger for vascular dementia (aHR 1.10, 1.06–1.14) than Alzheimer's dementia (aHR 1.03, 1.00–1.06). Hazard ratios were larger for severe psoriasis (all‐cause aHR 1.32, 1.25–1.39; vascular aHR 1.58, 1.44–1.74; Alzheimer's aHR 1.11, 1.02–1.21).

**Interpretation:**

Long‐term risk of all‐cause dementia and vascular dementia, but not Alzheimer's dementia, was slightly higher in people with psoriasis, but absolute risk differences were small. Risks were more substantially raised with time since psoriasis diagnosis and in severe psoriasis compared to mild to moderate psoriasis, suggesting a potential dose–response relationship.

## Background

Psoriasis is a chronic inflammatory skin disease characterised by skin scale, redness and induration. Psoriasis is common, with estimates of prevalence of around 2%.^1^ Dementia is a general term for loss of cognitive function, including memory, thinking and judgement such that it interferes with daily living. Globally, the number of people living with dementia is projected to rise from 50 million in 2018 to 150 million by 2050.^2^ While around 45% of dementia cases are attributable to 14 modifiable risk factors,^3^ the cause of many cases is unknown.

There is substantial evidence linking higher levels of inflammatory markers with Alzheimer's disease (a specific form of dementia),^4^, ^5^ and vascular dementia.^6^ It is also possible that sleep, which is known to be disturbed in both psoriasis and dementia, may play a causal role or suggest a common pathway relating to circadian dysregulation.^7^, ^8^ Some observational studies show associations between chronic inflammatory conditions such as psoriasis and risk of Alzheimer's disease and other dementias,^9^, ^10^, ^11^, ^12^ although evidence is conflicting.^13^, ^14^, ^15^, ^16^ A recent systematic review investigated the association between psoriasis and Alzheimer's disease specifically.^17^ Again, evidence was conflicting; while four of the six cohort studies included in the review offered evidence that psoriasis increased Alzheimer's disease risk, one suggested that psoriasis reduced Alzheimer's disease risk and another (using UK primary care electronic health records) reported no evidence of association (HR 1.23, 95% CI 0.82, 1.85). The review concluded that “further studies should be conducted to find the explanation of the discrepancy in the results.”

Few existing studies have investigated how psoriasis severity and treatments influence risk (evidence of dementia increasing with increased severity of psoriasis would offer stronger evidence for a causal relationship), and most existing studies account for a limited set of potential confounders.^18^, ^19^ A better understanding of the relationship between psoriasis and dementia in studies designed to assess causality may help to identify specific inflammatory pathways as targets for dementia prevention. This information could lead to the introduction of targeted cognitive function monitoring in people with psoriasis or the development of new treatments to prevent or delay dementia in people with psoriasis.

Therefore, our study investigated the association between psoriasis and all‐cause dementia risk in a large population‐based cohort using routinely collected electronic health records from English primary and secondary care. We specifically investigated whether increasing psoriasis severity affected dementia risk.

## Methods

We conducted an age‐, sex‐ and primary care practice‐matched cohort study (April 1997 to March 2021) using primary care data from the Clinical Practice Research Datalink (CPRD) Aurum (March 2023 build).^20^ CPRD Aurum is a database of routinely collected electronic health records from primary care practices in England using EMIS® software, including over 20% of the UK population.^21^ We also used linked data from: (1) hospital admissions for NHS‐funded patients treated in English NHS trusts or by independent providers (Hospital Episode Statistics [HES]); (2) Office for National Statistics (ONS) mortality records (includes cause of death for all UK deaths); and (3) Index of Multiple Deprivation as a measure of socioeconomic deprivation at a small‐area level based on patient postcode.^22^, ^23^, ^24^

Our study was approved by the London School of Hygiene and Tropical Medicine's Research Ethics Committee (Reference: 29636) and by CPRD's independent scientific advisory committee (Protocol number: 23_002911).

All computer code and morbidity code lists used to define all study variables are available for download (https://zenodo.org/doi/10.5281/zenodo.13759971).

### Study population

Individuals were eligible for inclusion in our study if they were aged 40 years or over, eligible for linkage to hospital admissions and death data, registered with Aurum practices during the study period (1997–2021), and with at least 12‐months' registration prior to study entry (for reliable capture of baseline health status). We restricted to adults aged 40 years and over as dementia is unusual in early life, and psoriasis has two peaks of onset in adulthood at ages 20–30 and 50–60 years, with most affected individuals presenting before age 35.^25^, ^26^ We excluded individuals with a history of dementia prior to study entry.

We identified a cohort of individuals with psoriasis based on a validated approach requiring a record of at least one diagnostic code for psoriasis in primary or secondary care (Fig. S1).^27^ We used SNOMED‐CT morbidity coding in primary care, and International Classification of Diseases, version 10 (ICD‐10) morbidity coding (recorded in any diagnostic position of any episode of a hospital admission) in secondary care.

We followed individuals with psoriasis from the latest of the following (hereafter referred to as the *index date*): first record of a diagnostic morbidity code for psoriasis (recorded in primary or secondary care); practice registration plus 1 year; study start (1^st^ April 1997), or 40^th^ birthday. The psoriasis cohort included both incident psoriasis cases (i.e., those entering the cohort on the date of their first record of a psoriasis diagnosis), and existing psoriasis cases (i.e., those whose first record of a diagnostic code for psoriasis occurred before any of the following: one year from practice registration date, study start, or their 40th birthday).

We randomly matched (without replacement) on age (within 2 years), sex and primary care practice up to five individuals without psoriasis for every individual with psoriasis in calendar date order (i.e., individuals in the matched cohorts were assigned first to individuals with psoriasis with earliest cohort entry to avoid time‐related bias). Individuals without psoriasis entered the cohort on the same date (i.e., index date) as the matched psoriasis‐exposed individual.

Participants were followed until the earliest of: dementia diagnosis, death, end of registration, no further data from practice, end of study (31st March 2021), or, for matched comparators only, a record of a diagnostic code for psoriasis. Individuals without psoriasis who received a subsequent record of a diagnostic code for psoriasis (recorded in primary or secondary care) after cohort entry (index date) were censored from the comparison cohort and became eligible for inclusion in the psoriasis cohort. We excluded individuals with less than 1 day of follow‐up.

### Outcome

We defined our dementia outcome as the earliest record of a diagnostic code for dementia recorded: in primary care (CPRD), and in secondary care as part of a hospital admission (HES).^28^, ^29^, ^30^ We investigated all‐cause dementia as our main outcome and dementia subtypes as secondary outcomes. Dementia subtypes (Alzheimer's disease (AD) and vascular dementia) were identified using morbidity codes recorded in primary care, or as part of a hospital admission.

As some dementia subtypes are unlikely to be caused by psoriasis (e.g. dementia related to drugs, alcohol, infection [e.g. HIV, Creutzfeldt‐Jakob], trauma, or inherited dementias [e.g. Huntington's]) we conducted a sensitivity analysis excluding these subtypes from our all‐cause dementia outcome.

### Covariates

We used a directed acyclic graph (DAG) to help identify potential confounders and mediators (i.e. on the causal pathway) of the relationship between psoriasis and dementia. We considered the following variables as potential covariates: age, sex, calendar period, socioeconomic deprivation, ethnicity, lifestyle factors (smoking, obesity, harmful alcohol use), chronic comorbidities (chronic liver disease, chronic lung disease, cerebrovascular disease, cardiovascular disease, diabetes mellitus, depression, hypertension, chronic kidney disease, high cholesterol, hearing loss, HIV‐positive status, rheumatoid arthritis, multiple sclerosis, frailty, psoriatic arthropathy) and sleep problems.

All covariates were defined based on records on or before cohort entry (except smoking, which was defined pragmatically based on the records closest to cohort entry).

We considered the calendar period (categorised as: 1997–2003, 2004–2011; 2012–2013; 2014–2015, 2016–2019, 2020–2021) to account for changes in clinical, diagnostic and administrative practices over the study period that may have influenced measurement of exposure, outcomes and other covariates.

We used quintiles of the index of multiple deprivation (IMD) as a proxy for socioeconomic deprivation. We used individual‐level IMD supplemented with practice‐level measures for those with missing individual‐level data.

Ethnicity was identified based on a previously validated algorithm using primary care records.^31^, ^32^

We defined smoking status and being overweight/obese (i.e., body mass index [BMI] suggesting individuals were overweight/obese) based on primary care records for these measures, using the status recorded closest to index date, based on a previously defined algorithm.^33^ We defined a binary overweight/obese variable based on a BMI of 25 kg/m^2^ or more.

We defined harmful alcohol use based on primary care morbidity codes suggesting harmful or heavy alcohol use (including alcohol dependency codes and codes related to physical/psychological harm related to alcohol use) or a prescription for drugs used to maintain abstinence (acamprosate, disulfiram, nalmefene). Individuals were defined as harmful alcohol users on the date of the first record of a relevant morbidity code or prescription.

We defined most chronic comorbidities (except chronic kidney disease and high cholesterol) based on morbidity coding in primary or secondary care. Individuals were regarded as having one of these diagnoses from the earliest record of a relevant diagnostic code.

As coded chronic kidney disease (CKD) underestimates CKD in primary care,^34^ we defined CKD status using both morbidity coding (defined based on the earliest record of a relevant diagnostic code) and estimated glomerular filtration rate (eGFR) based on serum creatinine test results. We defined CKD using test results as an eGFR <60 mL/min/1.73 m^2^ (i.e. Stage 3 CKD and above).

We defined high cholesterol based on morbidity coding or test results.

We defined problems with sleep using primary care morbidity codes suggesting sleep problems, prescriptions for drugs used exclusively to manage sleep problems (e.g. Zopiclone) and prescriptions for benzodiazepines where dosing instructions suggest use at night only (as when benzodiazepines are used to manage anxiety, they are usually prescribed for daytime use). Individuals were defined as having a sleeping problem from the first recorded morbidity code or sleeping tablet prescription. It is unlikely that this definition will be able to reliably capture all sleep problems, therefore we only adjusted for sleep in a sensitivity analysis.

Further details of study variable definitions are available in Text S1.

### Statistical analyses

We presented initial descriptive characteristics of cohorts with and without psoriasis. We explored the potential for ascertainment bias by describing the median (IQR) of consultations in the year before cohort entry in those with and without psoriasis. We then calculated age‐ and sex‐adjusted rates of incident dementia and dementia subtypes in those with and without psoriasis (age‐ and sex‐adjusted rates for the psoriasis‐exposed group, and adjusted absolute rate differences comparing rate in those with and without psoriasis).

#### Main analyses

We used stratified Cox regression (stratified by matched set and adjusted for potential confounders) to estimate hazard ratios (95%) comparing the hazard of dementia in those with and without psoriasis. We used our directed acyclic graph to inform covariate selection and causal model building. We used sequential models to account for the complexity of the potential relationship between psoriasis and dementia: (1) minimally adjusted including only the main exposure variable (psoriasis) and implicitly adjusting for age, sex and general practice (due to underlying timescale and matching variables); (2) additionally adjusting for deprivation and calendar period; (3) additionally adjusting for chronic comorbidities where the existing literature supports the possibility that they may act as confounders (i.e., chronic liver disease, chronic lung disease, cardiovascular disease, cerebrovascular disease, diabetes mellitus, depression, high cholesterol, hypertension and CKD); (4) additionally adjusting for ethnicity (included in a separate model to limit selection bias due to excluding those with missing ethnicity data complete‐case analysis). The direction of association between psoriasis and many of the chronic comorbidities is unclear. There is some evidence to suggest the inflammatory processes involved in psoriasis (or psoriasis treatments) may exacerbate some of these conditions, which would suggest they are mediators on the causal pathway.^35^ However, newer Mendelian randomisation studies also provide mixed evidence that some of these may be causally associated with the development of psoriasis.^36^ Therefore, we ran models with and without chronic comorbidities as covariates.

#### Sensitivity analyses

We repeated our main analyses in a series of sensitivity analyses to assess the robustness of our findings: (1) excluding practice non‐attenders; (2) ending the study on 1st March 2020 to explore the impact of including pandemic time in our study; (3) excluding specific dementia subtypes that are unlikely to be related to psoriasis (e.g. Creutzfeldt‐Jakob); (4) additionally adjusting for smoking status and overweight/obese (as these were omitted from primary models because of the proportion of missing values for these variables were high); (5) additionally adjusting for comorbidities where the evidence that they may act is confounder is less clear (e.g. hearing loss, HIV‐positive status); (6) additionally adjusting for sleep problems; (7) time‐updating covariates through follow‐up to explore the potential for these variables mediating the relationship between psoriasis and dementia; and (8) restricting to individuals with incident psoriasis (full rationale for these sensitivity analyses in Table S1).

#### Secondary analyses

In separate secondary analyses, we investigated whether the association between psoriasis and dementia was: (1) different with varying lengths of time since initial psoriasis diagnosis (analysis limited to individuals with incident psoriasis, with an interaction between follow‐up time in 5‐year bands and psoriasis); (2) more pronounced in individuals with severe psoriasis (by redefining exposure as mild–moderate psoriasis and severe psoriasis—defined based on primary care records for drug prescribing (Text S2)—compared to individuals without psoriasis); (3) modified by frailty (as the measure of frailty that we used was developed and validated in people aged 65–95 years, we restricted this analysis to those aged 65 and older); (4) mediated by psoriatic arthropathy. Psoriatic arthropathy may mediate the relationship between psoriasis and dementia because: (1) The burden of inflammation is likely to be greater in those with psoriatic arthropathy and psoriasis (and consequently a greater risk of dementia if there is a link between inflammation and dementia) and (2) Individuals with psoriatic arthropathy and psoriasis are more likely to receive a systemic drug and are also more likely to be obese.

## Results

**Figure 1:**
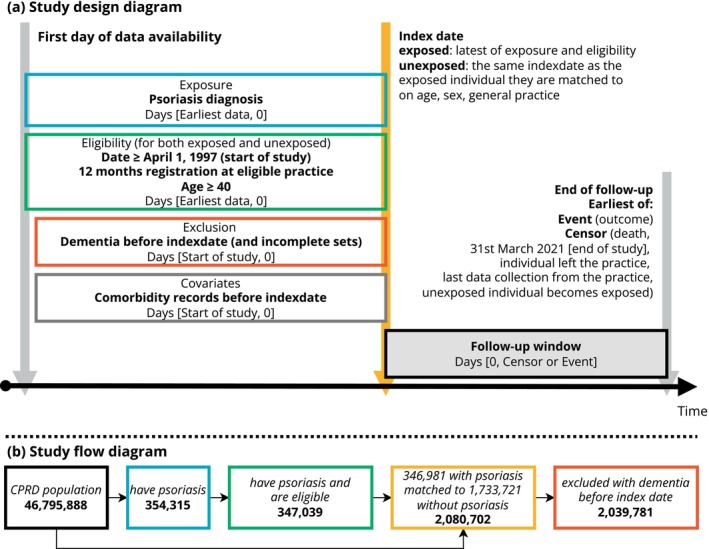
Flowchart illustrating identification of participants. Numbers of people with and without psoriasis do not sum to the total number of individuals included in each cohort. Individuals who were subsequently diagnosed with psoriasis could be included in the matched comparison cohort up until the date of their first psoriasis diagnosis.

We identified 346,981 individuals with psoriasis matched with 1,733,721 individuals without (Fig. 1). Individuals were followed up on average (median) 6.9 years (IQR 3.2–12.3) in the psoriasis group and 6.6 years (IQR 2.9–12.0) in the comparator cohort (Table 1). Cohorts with and without psoriasis were balanced on age (82% aged 40–69 in both groups), sex (53% female in both groups) and deprivation. The proportion of individuals recorded with chronic comorbidities was slightly higher for most comorbidities in those with psoriasis than their matched comparators (e.g. cardiovascular disease: psoriasis, 11%, comparators, 9%). Those with psoriasis had more consultations in the year before cohort entry (median [IQR]: psoriasis 12 [6–22], comparators 7 [3–15]).

**Table 1:** Study cohort characteristics at cohort entry.

	With psoriasis (*n* = 346,981)	Without psoriasis (*n* = 1,733,721)
Follow‐up		
Total person‐years	2,840,228	13,753,442
Median (IQR) (years)	6.9 (3.2, 12.3)	6.6 (2.9, 12.0)
Female	183,491 (53%)	916,907 (53%)
Age		
40–49	140,911 (41%)	704,354 (41%)
50–59	77,839 (22%)	389,172 (22%)
60–69	66,436 (19%)	332,133 (19%)
70–79	42,569 (12%)	212,739 (12%)
80–89	16,843 (4.9%)	84,084 (4.8%)
90–99	2359 (0.7%)	11,160 (0.6%)
100+	24 (<0.1%)	79 (<0.1%)
Index of multiple deprivation quintile		
1, least deprived	77,178 (22%)	398,819 (23%)
2	75,431 (22%)	380,816 (22%)
3	67,893 (20%)	336,145 (19%)
4	64,838 (19%)	318,431 (18%)
5, most deprived	61,514 (18%)	298,627 (17%)
Missing	127 (<0.1%)	883 (<0.1%)
Ethnicity		
White	222,054 (64%)	1,042,633 (60%)
South Asian	9913 (2.9%)	53,615 (3.1%)
Black	1849 (0.5%)	28,585 (1.6%)
Other/mixed	49,993 (14%)	236,587 (14%)
Missing	57,478 (17%)	340,767 (20%)
Body mass index		
Not overweight/obese (BMI < 25 kg/m^2^)	85,125 (25%)	456,753 (26%)
Overweight/obese (BMI > =25 kg/m^2^)	179,543 (52%)	795,410 (46%)
Missing	82,313 (24%)	481,558 (28%)
Smoking		
Non‐smoker	127,476 (37%)	745,650 (43%)
Current‐ or ex‐smoker	172,844 (50%)	688,319 (40%)
Missing	46,661 (13%)	299,752 (17%)
Harmful alcohol use	15,009 (4.3%)	48,403 (2.8%)
Chronic comorbidities		
Chronic liver disease	3079 (0.9%)	9388 (0.5%)
Chronic lung disease	18,489 (5.3%)	67,340 (3.9%)
Cardiovascular disease	37,724 (11%)	161,833 (9.3%)
Cerebrovascular disease	11,088 (3.2%)	48,257 (2.8%)
Diabetes mellitus	30,189 (8.7%)	125,437 (7.2%)
Depression	84,747 (24%)	338,515 (20%)
High cholesterol	174,495 (50%)	813,357 (47%)
Hypertension	88,497 (26%)	389,791 (22%)
Chronic kidney disease	35,295 (10%)	165,979 (9.6%)
Median (IQR) consultations in year before cohort entry	12 (6, 22)	7 (3, 15)

Data are *n* (%) unless otherwise specified.

Compared to the overall cohort, those with missing ethnicity, smoking status, or BMI were somewhat more likely to be male, older, less deprived and had somewhat fewer comorbidities recorded (Table S2).

### Main analysis

**Figure 2:**
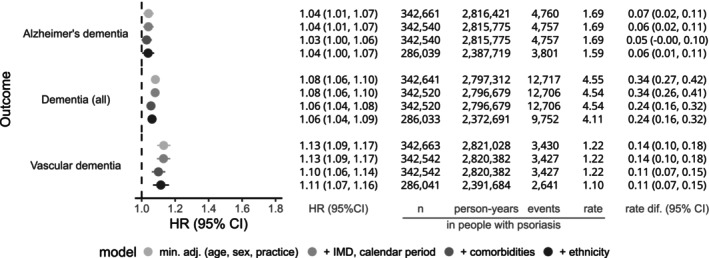
Hazard ratios (95% CI) comparing hazards of dementia in people with psoriasis to a matched population without. Fitted to individuals with complete data for all variables included in each model and from valid matched sets (matched sets including one psoriasis‐exposed individual and at least one unexposed comparator without psoriasis). Number of individuals (n), person‐years, number of outcomes (events) and absolute rate per 1000 person‐years are given for people with psoriasis. The rate difference per 1000 person‐years (with 95% confidence intervals) between people with and without psoriasis is calculated as the rate in those with psoriasis minus the estimated rate in those without psoriasis (rate in those with psoriasis × [1/hazard ratio]). *Models*: *1. (min. adj. [age, sex, practice])*: Implicitly adjusted for age, sex, and general practice (due to underlying timescale and matching); *2. (+ IMD, calendar period)*: Additionally adjusted for deprivation via quintiles of the Index of Multiple Deprivation (IMD) and calendar period (1997–2003, 2004–2011, 2012–2013, 2014–2015, 2016–2019, 2020–2021); *3. (+ comorbidities)*: Additionally adjusting for chronic comorbidities where we feel there is convincing evidence that they act as confounders (i.e., chronic liver disease, chronic lung disease, cardiovascular disease, cerebrovascular disease, diabetes mellitus, depression, high cholesterol, hypertension, and CKD); *4. (+ ethnicity)*: Additionally adjusted for ethnicity.

In individuals with psoriasis (*n* = 342,641), 12,717 dementia events occurred (4760 Alzheimer's dementia, 3430 vascular dementia, 4527 not specified or other dementia) in 2,797,312 person‐years (a rate of 4.55 per 1000 person‐years). Individuals with psoriasis had a slightly higher hazard of developing dementia compared to matched comparators (crude HR 1.08, 95% CI 1.06–1.10; adjusted HR [deprivation, calendar period, comorbidity] 1.06, 95% CI 1.04–1.08; adjusted absolute rate difference 0.24, 95% CI 0.16–0.32 per 1000 person‐years). HRs were smaller and crossed the null for Alzheimer's dementia (adjusted HR 1.03, 95% CI 1.00–1.06), and were larger for vascular dementia (adjusted HR 1.10, 95% CI 1.06–1.14) (Fig. 2).

### Sensitivity analyses

Results from all sensitivity analyses where the cohort composition was changed and/or additional variables (including frailty) were adjusted for were generally similar to those from the main analysis. Effect estimates were slightly attenuated when adjusting models for BMI and smoking (adjusted HR 1.03 [1.00–1.05]), and when adjusting for time‐updated comorbidity status (adjusted HR for dementia 1.03 [1.00–1.06]) (Table S1).

### Secondary analyses

#### Follow‐up time

**Figure 3:**
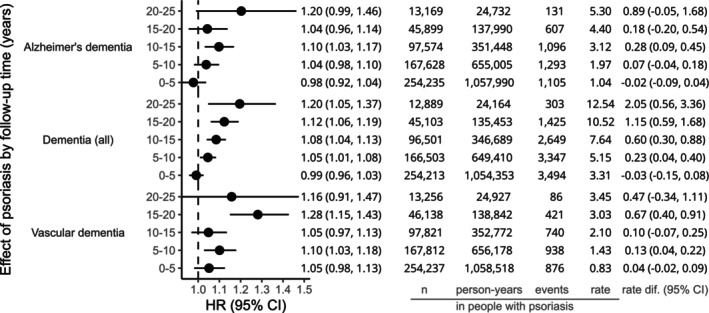
Hazard ratios (95% CI) comparing hazards of dementia in people with incident psoriasis to a matched population without, including interaction with follow‐up time in 5‐year bands. Results are from a cohort of incident psoriasis and comorbidity‐adjusted models (adjusted for age, sex, and general practice due to underlying timescale and matching, and quintiles of the Index of Multiple Deprivation, calendar period, chronic liver disease, chronic lung disease, cardiovascular disease, cerebrovascular disease, diabetes mellitus, depression, high cholesterol, hypertension, and CKD). Fitted to individuals with complete data for all variables included in each model and from valid matched sets (matched sets including one psoriasis‐exposed individual and at least one unexposed comparator without psoriasis). Number of individuals (*n*), person‐years, number of outcomes (events) and absolute rate per 1000 person‐years are given for people with psoriasis. The rate difference per 1000 person‐years (with 95% confidence intervals) between people with and without psoriasis is calculated as the rate in those with psoriasis minus the estimated rate in those without psoriasis (rate in those with psoriasis × [1/hazard ratio]).

After limiting to those with incident psoriasis, hazard ratios for dementia increased with time since psoriasis diagnosis (0–5 years: 0.99 [0.96–1.03]; 5–10 years: 1.05 [1.01–1.08]; 10–15 years: 1.08 [1.04–1.13]; 15–20 years 1.12 [1.06–1.19]; 20–25 years 1.20 [1.05–1.37]). For vascular dementia, we saw the largest hazard ratio in the 15‐20‐year period (1.28 [1.15–1.43]). For Alzheimer's dementia, confidence intervals overlapped across all time periods (Fig. 3).

#### Psoriasis severity

**Figure 4:**
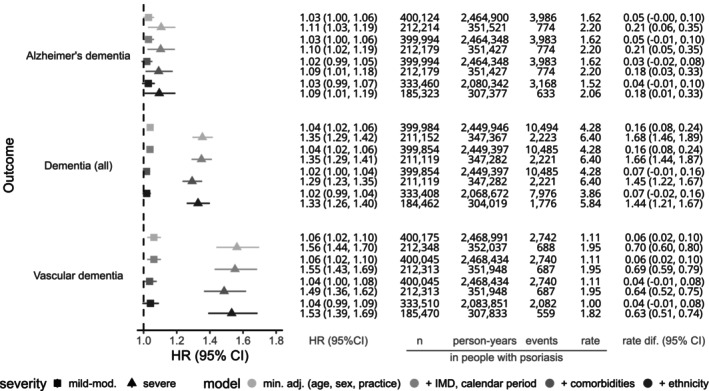
Hazard ratios (95% CI) comparing hazards of dementia in people with mild/moderate psoriasis, or severe psoriasis to a matched population without. Fitted to individuals with complete data for all variables included in each model and from valid matched sets (matched sets including one psoriasis‐exposed individual and at least one unexposed comparator without psoriasis). Number of individuals (*n*), person‐years, number of outcomes (events) and absolute rate per 1000 person‐years are given for people with psoriasis. The rate difference per 1000 person‐years between people with and without psoriasis is calculated as the rate in those with psoriasis minus the estimated rate in those without psoriasis (rate in those with psoriasis × [1/hazard ratio]). *Models*: *1. (min. adj. [age, sex, practice])*: Implicitly adjusted for age, sex, and general practice (due to underlying timescale and matching); *2. (+ IMD, calendar period)*: Additionally adjusted for deprivation via quintiles of the Index of Multiple Deprivation (IMD) and calendar period (1997–2003, 2004–2011, 2012–2013, 2014–2015, 2016–2019, 2020–2021); *3. (+ comorbidities)*: Additionally adjusting for chronic comorbidities where we feel there is convincing evidence that they act as confounders (i.e., chronic liver disease, chronic lung disease, cardiovascular disease, cerebrovascular disease, diabetes mellitus, depression, high cholesterol, hypertension, and CKD); *4. (+ ethnicity)*: Additionally adjusted for ethnicity.

The adjusted HR (95% CI) comparing those with mild psoriasis to matched comparators without psoriasis was 1.02 (1.00–1.04), and for severe psoriasis was 1.29 (1.23–1.35). HRs for severe psoriasis compared to matched comparators were larger for vascular dementia (1.49 [1.36–1.62]) than for Alzheimer's dementia (1.09 [1.01–1.18]) (Fig. 4). In a sensitivity analysis where we matched individuals with severe psoriasis to people without psoriasis (instead of using time‐updated psoriasis severity status within existing matched sets), effect estimates for severe psoriasis were somewhat smaller (e.g. comorbidity‐adjusted HR 1.23 [95% CI 1.16–1.29] for dementia, 1.45 [1.32–1.59] for vascular dementia, 1.11 [1.02–1.21] for Alzheimer's dementia) (Table S3).

#### Frailty

In our secondary analysis of people aged 65 years and over, we found the association between psoriasis and dementia was smaller in the mild, moderate and severe frailty groups, compared to those who were not frail, but confidence intervals overlapped (Table S4).

#### Psoriatic arthropathy

Additionally adjusting analyses for time‐updated psoriatic arthritis status did not considerably change effect estimates (Table S5).

## Discussion

We found people with psoriasis were at increased dementia risk, although relative risk (1.06 [1.04–1.08]) and absolute rate differences were small (24 per 100,000 person‐years). The magnitude of increased risk was higher for vascular dementia (1.10 [1.06–1.14]). However, there was no evidence of increased Alzheimer's dementia risk (1.03 [1.00–1.06]). Hazard ratios increased with time since the first psoriasis diagnosis. People with severe psoriasis had considerably increased vascular dementia risk (1.49 [1.36–1.62]), but not Alzheimer's dementia (1.09 [1.01–1.18]). Results were consistent across a range of sensitivity analyses. In summary, our findings suggest that, compared to the general population, people with psoriasis, especially with severe psoriasis, may be at long‐term increased risk of vascular, but not Alzheimer's, dementia.

The internal validity of our study was, like other observational studies, potentially limited by selection and information bias and residual confounding. Our study was population‐based and not restricted to specific demographic, hospital, or insurance groups. Hence, selection bias is not a major concern.

There may be misclassification in our dementia definition. Up to 40% of people living with dementia may not have a diagnosis.^37^ In addition, we relied on individuals consulting for, and having their condition recorded in electronic health records with a relevant morbidity code. However, using linked hospital admissions data to increase ascertainment was a strength.

Dementia ascertainment may be greater in people with psoriasis due to increased healthcare contact (reflected in a higher median consultation count in the year before cohort entry). However, a sensitivity analysis restricted to those with at least one consultation with their GP in the year before cohort entry to exclude practice non‐attenders was consistent with our main results, although the possibility of detection bias remains.

There may also be a misclassification of dementia subtype. Almost a third of dementia outcomes were classified as neither Alzheimer's nor vascular dementia. It is likely that some of these would constitute cases of Alzheimer's or vascular dementia, for example, when an individual's dementia subtype is not, or only later, classified. Clinical diagnosis of dementia subtype can also be inaccurate.^38^

Of the 14 main modifiable risk factors for dementia,^3^ we captured smoking, excessive alcohol consumption, hypertension, obesity, diabetes, hearing loss, depression and high cholesterol. Vision loss, physical inactivity (captured as activity limitation) and social isolation (captured as social vulnerability) were part of our frailty definition. We did not capture education and air pollution as these are not routinely recorded by GPs. We did not capture head injury as this was unlikely to be related to psoriasis. We captured additional factors that may confound or mediate the association between psoriasis and dementia, and we aimed to mitigate misclassification due to variability in coding practices via adjustment for calendar period (to account for temporal changes in diagnostic and coding practices). Nevertheless, the possibility of residual confounding remains.

We saw missing smoking and BMI status of between 13 and 28% (depending on exposure status and variable). Due to the high proportion of missing data, we only included overweight/obese and smoking status in a sensitivity analysis (as limiting analyses to those with complete smoking and BMI data may preferentially include those with both exposure [psoriasis] and outcome [dementia]), where effect estimates were somewhat attenuated compared to our main analysis.

We used two approaches to estimate hazard ratios, each with different strengths. First, we estimated hazard ratios across the entire follow‐up time in a cohort including existing and newly‐diagnosed psoriasis to maximise psoriasis capture (with the onset of many cases at a lower age than our study population of over 40s). Second, to assess the impact of time with psoriasis, we used a cohort of only newly diagnosed cases and estimated hazard ratios in 5‐year follow‐up time bands. The exact onset date of relapsing–remitting conditions like psoriasis cannot always be accurately captured in routinely collected data, meaning our cohort of newly‐diagnosed psoriasis may have included some with pre‐existing psoriasis. However, as would be expected if psoriasis‐related dementia risk increases with time, we found no increased dementia risk within the first 5 years after newly diagnosed psoriasis.

Ideally, our study would aim to investigate a potential causal relationship between psoriasis and dementia. However, we need to be cautious about assessing causality given the complex relationship between psoriasis and dementia, and the potential confounders and mediators of the relationship. In many instances, the clinical reality means that some of the covariates we assessed could act as both confounders and mediators, and the direction of association between important variables may be in both directions (making relationships difficult to capture in a DAG). In our main analysis using a cohort of incident and prevalent psoriasis we were also unable to reliably assess whether some covariates occurred before or after psoriasis exposure. We explored some of the complexity of the relationship between exposure and outcome using a sequential modelling strategy, where we added variables that may act as mediators in later models, and found effect estimates were consistent with the main results. Our findings from a sensitivity analysis limiting our study population to individuals with new‐onset psoriasis and their matched comparators were also broadly consistent with those of our main analysis.

Our psoriasis severity definition may misclassify individuals with severe psoriasis as less severe disease if they refuse medical therapy. Misclassification of psoriasis status or severity may over‐ or underestimate the real association between psoriasis severity and dementia because early symptoms of dementia could influence diagnostic and treatment preferences. However, general practitioners will record dementia diagnoses independently and prospectively, so it is likely that reverse causality will affect all study participants equally regardless of psoriasis status (i.e., nondifferential misclassification, suggesting a bias toward null rather than spurious association). A further limitation of our psoriasis severity definition was that we were unable to capture symptom reduction or resolution (absence of records for psoriasis does not necessarily mean absence in symptoms). Consequently, we considered individuals as having severe disease from the date they meet the definition and may therefore wrongly classify people as having severe psoriasis when their symptoms have reduced or resolved, which may have diluted the effect of psoriasis severity on dementia and biasing our effect estimate to null.

Our psoriasis severity definition was based on psoriasis therapies. Psoriasis therapies may themselves affect the risk of dementia. However, with most psoriasis therapies reducing inflammation, it is possible that they actually reduce the risk of vascular dementia, meaning our results for severe psoriasis and vascular dementia may be underestimates.

As we did not have access to records of hospital‐delivered therapies (targeted drugs for psoriasis are predominantly delivered in secondary care) we may have missed a small number of individuals with severe psoriasis whose condition is solely managed in secondary care. However, we expect that these individuals will have been prescribed other severe‐psoriasis therapies in primary care prior to being managed with targeted biologics.

Our findings on the risk of dementia (comorbidity‐adjusted HR 1.06 [1.04–1.08]) and Alzheimer's dementia (1.03 [1.00–1.06]) are similar to previous large population‐based studies. A study in the Korean National Health Insurance System database (535,927 with psoriasis) found a hazard ratio of 1.09 (95% CI 1.07–1.12) for Alzheimer's dementia.^9^ A study in the Taiwan National Health Insurance Research Database (111,825 with psoriasis) found a hazard ratio of 1.02 (0.96–1.09) for all‐cause dementia.^15^ In both studies, and ours, such weakly increased risks may be fully explained by residual confounding or bias related to routinely collected data or observational study design. However, our study also offers a comparison between different types of dementia. The larger effect estimates seen for vascular dementia compared to Alzheimer's dementia, especially in those with severe psoriasis, make it less likely that an increased risk of vascular dementia can be fully explained by bias or confounding. Our findings of a greater risk of vascular dementia are also supported by previous work providing evidence of an association between psoriasis and vascular disease.^37^, ^38^, ^39^ However, the evidence of an association between other immune mediated inflammatory diseases (such as rheumatoid arthritis) and dementia is mixed.^40^ Thus, further research is needed to clarify the role of chronic systemic inflammation as a driver of psoriasis‐associated dementia risk.

Reassuringly, our study suggests it is unlikely people with psoriasis are at increased risk of Alzheimer's disease compared to the general population (or at least any increased risk is likely to be very small). While the finding of increased risk of all‐cause dementia was unlikely due to chance, the increase in risk was again very small and potentially not clinically important, or could be explained through residual bias or confounding. However, there may be some long‐term increased risk of vascular dementia, especially with more severe psoriasis, although absolute rate differences were small. Future research could use more granular psoriasis severity measures to more precisely identify those at increased risk and data with longer available follow‐up time could be used to further investigate the time it takes for psoriasis‐related dementia risk to develop.

## Author Contributions

KEM, SML, CWG, JM and SLC were involved in the development of the study. SLC, JM, KM, SML and CWG contributed to the development of the code lists that defined the variables used in the study. JM was responsible for data management and statistical analyses. KEM and JM wrote the first paper draft. All authors contributed to and approved the final manuscript.

## Funding Information

The study was funded by Open Philanthropy. CWG is supported by a Wellcome Career Development Award (225868/Z/22/Z). The study funder played no role in the study design; collection, analysis, and interpretation of data; in the writing of the report; and in the decision to submit the article for publication. All researchers were independent from funders and all authors had full access to the results of the analyses. All authors take responsibility for the integrity of the data and the accuracy of the data analysis.

## Conflict of Interest

All authors have completed the ICMJE uniform disclosure form (www.icmje.org/coi_disclosure.pdf). KEM reports personal fees from AMGEN, outside the submitted work. All other authors have nothing to disclose.

## Consent Statement

Consent was not necessary for this study because the Clinical Practice Research Datalink provides anonymised data to researchers.

## Transparency Statement

The lead authors (the manuscript's guarantor) JM and KEM, affirm that the manuscript is an honest, accurate and transparent account of the study being reported; that no important aspects of the study have been omitted; and that any discrepancies from the study as originally planned (and, if relevant, registered) have been explained.

## Supporting information


Appendix S1.

## Data Availability

No additional unpublished data are available as this study used existing data from CPRD that is only accessible to researchers with approved protocols. All data management and analysis computer code is available via zenodo (https://zenodo.org/doi/10.5281/zenodo.13759971). All code is shared without investigator support. Our study protocol is available as additional online‐only Supplementary Material.
