# Supplementary material for: Psoriasis and dementia: A population‐based matched cohort study of adults in England
Source: Ann Clin Transl Neurol. 2025 Jan 1;12(2):393–404. doi: 10.1002/acn3.52283 (PMC11822786; doi:10.1002/acn3.52283)
Supplement: Supplementary file 1 — Appendix S1. [file ACN3-12-393-s001.doc]

# Supplementary material

## **Text S1:** Covariate definitions

We considered **calendar period** to account for changes in clinical, diagnostic, and administrative practices over the study period that may have influenced the measurement of exposure, outcomes, and other covariates. We categorised calendar period as: 1997–2003, 2004–2011; 2012–2013; 2014–2015, 2016–2019, and 2020–2021. We used finer bands towards the end of the study period to account for the disruption in use and delivery of health care as a result of the COVID-19 pandemic.^1^ In deciding on calendar period bands, we have also accounted for: the 2012 Prime Minister’s dementia challenge, governmental NHS mandate in 2016-2017 and 2018-2019, which included an objective to maintain a minimum diagnosis rate of two thirds for people with dementia, and the Dementia Identification Scheme to reward GP practices for improving dementia detection rates in 2014-2015.^2,3^

**Ethnicity** was identified based on a previously validated algorithm using primary care records.^4^ We also explored the effect of ethnicity in a sensitivity analysis restricting to individuals entering the cohorts form 2006 onwards when records became more complete following the introduction of remuneration for including ethnicity data in the Quality and Outcomes Framework).

We pragmatically defined **smoking status** and being **overweight/obese** (i.e., body mass index [BMI] suggesting individuals are overweight or obese) based on primary care records for these measures, using the status recorded closest to index date, based on a previously defined algorithm.^5^ We calculated BMI using the most recent height and weight measures recorded before cohort entry date, and if these weren’t available we used the most recent recorded morbidity code for BMI (we did not use weight measures or BMI morbidity codes recorded after cohort entry date, as evidence indicates that the relationship between BMI and dementia over the life course is complex; with a relationship between being overweight/obese in middle age and dementia, but individuals being more likely to be underweight once dementia is established).^6^ We defined a binary overweight/obese variable based on a BMI of 25 kg/m^2^ or more.

We defined **harmful alcohol use** based on primary care morbidity codes suggesting harmful or heavy alcohol use (including alcohol dependency codes and codes related to physical/psychological harm related to alcohol use) or a prescription for drugs used to maintain abstinence (acamprosate, disulfiram, or nalmefene). Individuals were defined as harmful alcohol users on the date of the first record of a relevant morbidity code or prescription.

We defined most **chronic comorbidities** based on morbidity coding in primary or secondary care. Comorbidities defined this way will include: chronic liver disease, chronic lung disease (including: chronic obstructive pulmonary disease and interstitial lung diseases), cerebrovascular disease (including stroke and transient ischaemic attack), cardiovascular disease (including: ischaemic heart disease, cardiac failure, arrhythmia), diabetes mellitus, depression, hypertension, hearing loss, HIV positive status, rheumatoid arthritis, inflammatory bowel disease, multiple sclerosis, psoriatic arthropathy, and asthma. Individuals were regarded as having one of these diagnoses from the earliest record of a relevant diagnostic code. Diabetes mellitus was defined as a binary variable, based on individuals never/ever having a record of any type of diabetes and not classified by type.

As coded **chronic kidney disease** (CKD) underestimates CKD in primary care,^7^ we defined CKD status using both morbidity coding (defined based on the earliest record of a relevant diagnostic code) and estimated glomerular filtration rate (eGFR) based on serum creatinine test results. We defined CKD using test results based on an eGFR <60 mL/min/1.73 m2 (i.e., Stage 3 CKD and above) calculated from the most recent serum creatinine result recorded in the 12 months prior to cohort entry (and time-updated with subsequent eGFR measures) using the Chronic Kidney Disease Epidemiology Collaboration (CKD-EPI) equation (disregarding ethnicity in the calculation).^8^ Individuals with no morbidity code for CKD, and no recorded serum creatinine result suggesting CKD, were assumed to have no CKD.

We defined **high cholestero**l based on morbidity coding or test results.

We defined **problems with sleep** using primary care morbidity codes suggesting sleep problems, prescriptions for drugs used exclusively to manage sleep problems (e.g., Zopiclone), and prescriptions for benzodiazepines where dosing instructions suggest use at night only (as when benzodiazepines are used to manage anxiety, they are usually prescribed for daytime use). Individuals were defined as having a sleeping problem from their first recorded morbidity code or sleeping tablet prescription. As we expected that this definition would be unlikely to reliably capture all sleep problems, we only used it in a sensitivity analysis.

In a secondary analysis, we used the electronic frailty index (eFrailty) as a measure of **frailty**.^9^ The eFrailty index uses primary care morbidity coding across 36 deficits (deficits include specific morbidities [e.g., arthritis, hypertension, Parkinsonism], symptoms [e.g., activity limitation, dizziness], social issues [e.g., vulnerability, care requirements]) to categorise individuals as fit, mildly frail, moderately frail, or severely frail. The index uses a cumulative model of equally weighted individual deficits to calculate a score between zero and one based on the proportion of deficits identified out of the full list of thirty-six. However, we excluded the ﻿‘memory and cognitive problems’ deficit as we feel this deficit represents symptoms closely related to our dementia outcome.

## **Text S2:** Psoriasis severity

In a secondary analysis, we investigated whether risk of dementia increased with psoriasis severity. We based our definition of severe psoriasis on an existing definition where severe psoriasis was defined using records for therapies used to treat psoriasis,^10^ and additionally included hospital admission for psoriasis (i.e., psoriasis defined in the first diagnostic position of any episode). We identified psoriasis therapies recorded in primary care (SNOMED-CT morbidity coded or prescription records). Psoriasis therapies included: phototherapy, methotrexate, azathioprine, cyclosporine, mycophenolate, acitretin, etretinate, hydroxycarbamide, and fumaric acid esters. Our psoriasis-severity definition is a well-accepted approach for defining severity in routinely collected electronic health data studies in psoriasis.^11,12^ However, our psoriasis-severity definition will miss hospital-prescribed (where most of these drugs are prescribed) targeted biologic therapies for psoriasis, but we assume that individuals managed with biologics will have been treated with other severe-psoriasis therapies,^13^ which will be prescribed in primary care, before they are managed with targeted biologics.

We updated psoriasis severity over time, that is, individuals were classified as having mild/moderate psoriasis until the date they first satisfied the requirements of the severe psoriasis definition. When (or if) an individual satisfied the requirements of the definition for severe psoriasis, they switched to the severe category and remain in it for the rest of follow-up.

In our secondary analysis of psoriasis severity, we reclassified exposure status as mild/moderate psoriasis or severe psoriasis, compared to the matched cohort without psoriasis.

People identified as having severe psoriasis during follow up may be more likely to have a dementia diagnosis recorded in the later period of their follow up (when they are classified as having severe disease) than the earlier period (when they are classified as having mild/moderate psoriasis). However, they will still be compared to the controls from the original matched sets. Individuals who develop severe psoriasis during follow up are consequently likely to contribute less follow up time than their comparators due to the design of the study. Therefore, in a follow up sensitivity analysis, rather than using the original matched sets (where individuals with psoriasis were matched to those without regardless of psoriasis severity status) as comparators, we re-matched individuals when they were classified as having severe psoriasis to those without psoriasis at the first time they meet our severe psoriasis definition.

## **References**

1 Mansfield KE, Mathur R, Tazare J, *et al.* Indirect acute effects of the COVID-19 pandemic on physical and mental health in the UK: a population-based study. *Lancet Digit Health* 2021; published online Feb. DOI:10.1016/s2589-7500(21)00017-0.

2 Warren-Gash C. Dementia risk prediction models: what do policy makers need to know? 2019 www.phgfoundation.org.

3 Parkin E, Baker C. Dementia: policy, services and statistics overview. 2021 https://commonslibrary.parliament.uk/research-briefings/sn07007/%0Ahttps://researchbriefings.files.parliament.uk/documents/SN07007/SN07007.pdf%0Ahttps://commonslibrary.parliament.uk/research-briefings/sn07007/%0Ahttp://researchbriefings.files.parliament.u.

4 Mathur R, Bhaskaran K, Chaturvedi N, *et al.* Completeness and usability of ethnicity data in UK-based primary care and hospital databases. *J Public Health (Oxf)* 2013; **36**: 684–92.

5 Bhaskaran K, Forbes HJ, Douglas I, Leon DA, Smeeth L. Representativeness and optimal use of body mass index (BMI) in the UK Clinical Practice Research Datalink (CPRD). *BMJ Open* 2013; **3**: e003389.

6 García-Ptacek S, Faxén-Irving G, Čermáková P, Eriksdotter M, Religa D. Body mass index in dementia. *Eur J Clin Nutr* 2014; **68**: 1204–9.

7 Iwagami M, Tomlinson LA, Mansfield KE, *et al.* Validity of estimated prevalence of decreased kidney function and renal replacement therapy from primary care electronic health records compared with national survey and registry data in the United Kingdom. *Nephrology Dialysis Transplantation* 2017; : 1–9.

8 Levey AS, Stevens LA, Schmid CH, *et al.* A new equation to estimate glomerular filtration rate. *Ann Intern Med* 2009; **150**: 604–12.

9 Clegg A, Bates C, Young J, *et al.* Development and validation of an electronic frailty index using routine primary care electronic health record data. *Age Ageing* 2016; **45**: 353–60.

10 Gelfand JM, Troxel AB, Lewis JD, *et al.* The Risk of Mortality in Patients With Psoriasis. *Arch Dermatol* 2007; **143**: 1493–9.

11 Yiu ZZN, Parisi R, Lunt M, *et al.* Risk of hospitalization and death due to infection in people with psoriasis: a population-based cohort study using the Clinical Practice Research Datalink*. *British Journal of Dermatology* 2021; **184**: 78–86.

12 Yeung H, Takeshita J, Mehta NN, *et al.* Psoriasis severity and the prevalence of major medical comorbidity: A population-based study. *JAMA Dermatol* 2013; **149**: 1173–9.

13 National Institute for Health and Clinical Excellence. Psoriasis : assessment and management. 2017.

14 Lutsey PL, Chen N, Mirabelli MC, *et al.* Impaired lung function, lung disease, and risk of incident dementia. *Am J Respir Crit Care Med* 2019; **199**: 1385–96.

15 Mleczko M, Gerkowicz A, Krasowska D. Chronic Inflammation as the Underlying Mechanism of the Development of Lung Diseases in Psoriasis: A Systematic Review. *Int J Mol Sci* 2022; **23**. DOI:10.3390/ijms23031767.

16 Kontopantelis E, Olier I, Planner C, *et al.* Primary care consultation rates among people with and without severe mental illness: A UK cohort study using the Clinical Practice Research Datalink. *BMJ Open* 2015; **5**: 1–10.

17 National Institute for Health and Care Excellence (NICE). Psoriasis: assessment and management. 2012 https://www.nice.org.uk/guidance/cg153/resources/psoriasis-assessment-and-management-pdf-35109629621701.

18 Manzo C, Castagna A, Ruberto C, Ruotolo G. Does a steroid dementia syndrome really exist? A brief narrative review of what the literature highlights about the relationship between glucocorticoids and cognition. *Geriatric Care* 2023; **8**. DOI:10.4081/gc.2022.10975.

## **Table S1:** Sensitivity analyses

| **Analysis** | **Description and justification** | **HR (95% CI) (comorbidity-adjusted unless otherwise specified)** |
| --- | --- | --- |
| **Main analysis** | **Included for comparison.** | **1.06 (1.04 – 1.09)** |
| Excluding practice non-attenders | Restricting to individuals with at least one consultation with their GP in the year before cohort entry to exclude practice non-attenders. | 1.05 (1.03 – 1.08) |
| Pandemic time | Our main analysis adjusted for calendar time, using finer bands in more recent time to account for the disruption in use and delivery of health care due to the COVID-19 pandemic. However, it is possible that our results may be biased by changes in consulting practices due to the pandemic. Consequently, to explore the impact of including pandemic time in our analyses, we repeated our analysis ending the study on 1st March 2020. | 1.05 (1.03 – 1.08) |
| Excluding specific dementia subtypes | Excluding dementia subtypes unlikely to be related to psoriasis from our all-cause dementia outcome: Some dementia subtypes are unlikely to be related to psoriasis (e.g., dementia related to drugs, alcohol, infection [e.g., HIV, Creutzfeld-Jakob], trauma, or inherited dementias [e.g., Huntington’s]) we conducted a sensitivity analysis excluding these subtypes from our all-cause dementia outcome. | 1.05 (1.03 – 1.08) |
| BMI and smoking status | BMI and smoking status may confound the association between psoriasis and dementia. Due to the large proportion of missing values, especially for BMI, we considered analyses additionally adjusting for BMI and smoking status as sensitivity analyses. In addition, BMI and smoking status may be collinear with comorbidities. | 1.03 (1.00-1.05) |
| Additional comorbidities | In a sensitivity analysis, we additionally adjusted for comorbidities where the evidence that they may act is confounder is less clear (i.e., hearing loss, HIV-positive status, rheumatoid arthritis, multiple sclerosis, inflammatory bowel disease).  We additionally adjusted for potential covariates identified during directed acyclic graph development whether there was less convincing evidence that they act as confounders. For example, evidence indicates that asthma is associated with dementia (outcome),^14^ but there is less convincing evidence linking psoriasis (exposure) to asthma.^15^ | 1.07 (1.05 – 1.09) |
| Adjusting for sleep problems | Disrupted sleep in both psoriasis and dementia may mediate the relationship or suggest a common pathway. However, general practitioners do not routinely record patients’ quality of sleep, so we do not believe that we can reliably capture a measure of sleep quality using routinely recorded data. Therefore, we felt that it would be inappropriate to adjust for sleep disturbances in our main analyses. However, we conducted a sensitivity analysis additionally adjusting for a record of a morbidity code for a sleep disturbance, or a prescription for a drug used to manage sleeping problems, as a means of beginning to explore the potential impact of sleep disturbance in the relationship between psoriasis and dementia. | 1.07 (1.05 – 1.09) |
| Time-updated covariates | In our main analysis, we defined covariates based on their status at baseline. In a sensitivity analysis we time updated the status of covariates defined by a record of a relevant morbidity code the first time the code was recorded, to explore the potential for covariates mediating the relationship between psoriasis exposure and dementia outcome. | 1.03 (1.00 – 1.06) |
| Incident psoriasis | Restricting to individuals with a diagnosis of newly-active psoriasis in the exposed cohort (exposed individuals defined as those who joined the cohort when they first fulfilled the study’s diagnostic criteria after the start of the study period) and their matched sets, in order to: 1) be more confident that the covariates captured at cohort entry precede the onset of psoriasis (exposure), so that they are less likely to be on the causal pathway between psoriasis and dementia; and 2) in order to capture a measure of duration of time experiencing inflammation due to psoriasis. | 1.07 (1.05 – 1.08) |
| Cohort entry from 2004 | We restricted to individuals entering the cohort from 2004 onwards to account for changes in **diagnostic and coding practices over time** – specifically those triggered by the introduction of the Quality and Outcomes Framework in 2004 – when coding of some variables is likely to be more complete. This is likely to be more important for dementia outcomes and some covariates.^16^ However, as there are no specific dermatology indicators in the Quality and Outcomes Framework, it is unlikely that psoriasis coding was affected. | 1.06 (1.04 – 1.09)  1.04 (1.02-1.07) when additionally adjusting for BMI and smoking |
| Cohort entry from 2006 | To examine whether including **ethnicity** as a covariate in the main analysis introduced selection bias (by restricting those with complete ethnicity data in a complete-case analysis) we restricted to a subset of individuals registered from 2006 onwards. Records for ethnicity became more complete following the introduction of remuneration for including ethnicity data in the Quality and Outcomes Framework.^4^ | 1.06 (1.04 – 1.09)  1.06 (1.04 – 1.09) when additionally adjusting for ethnicity |
| Adjusting for high-dose steroids | Additionally adjusting for high-dose oral steroid prescription: While systemic steroids are not recommended for the management of psoriasis,^17^ some clinicians may still prescribe them, and they may be more likely to be prescribed in individuals with psoriasis for concomitant psoriatic arthropathy. There is some limited evidence that glucocorticoids may cause a reversible cognitive decline.^18^ We therefore adjusted for ever having received a prescription for a high-dose oral glucocorticoid (>=20mg prednisolone equivalent dose per day) to investigate the potential for steroids mediating the relationship between psoriasis and dementia. | 1.07 (1.05 – 1.09) |

## **Table S2: ﻿**Characteristics of the study population at cohort entry, for the overall cohort and those included, and excluded, from complete case analyses for analyses adjusting for variables with missing data.

|  | **Overall cohort** | | **Included in analyses additionally adjusted for ethnicity** | | **Excluded from analyses adjusted for deprivation, ethnicity and calendar period due to missing ethnicity data** | | **Included in analyses additionally adjusted for lifestyle variables (smoking, harmful alcohol use, overweight/obese)** | | **Excluded from analyses additionally adjusted for lifestyle variables due to missing lifestyle data (smoking, or overweight/obese status)** | |
| --- | --- | --- | --- | --- | --- | --- | --- | --- | --- | --- |
|  | **With psoriasis**  (n = 346,981) | **Without psoriasis**  (n= 1,733,721) | **With psoriasis**  (n = 289,503 (100%)) | **Without psoriasis**  (n= 1,392,954 (100%)) | **With psoriasis**  (n = 57,478) | **Without psoriasis**  (n= 340,767) | **With psoriasis**  (n = 259,682) | **Without psoriasis**  (n= 1,224,536) | **With psoriasis**  (n = 87,299) | **Without psoriasis**  (n= 509,185) |
| **Follow-up** |  |  |  |  |  |  |  |  |  |  |
| Total person years | 2,840,228 | 13,753,443 | 2,407,240 | 11,291,274 | 432,988 | 2,462,169 | 1,801,706 | 8,079,028 | 1,038,521 | 5,674,415 |
| Median (IQR) years | 6.9 (3.2, 12.3) | 6.6 (2.9, 12.0) | 7.0 (3.2, 12.6) | 6.8 (3.0, 12.3) | 6.2 (2.8, 11.2) | 5.8 (2.6, 10.8) | 5.8 (2.8, 10.3) | 5.5 (2.5, 9.8) | 12 (6, 18) | 11 (5, 17) |
| **Female** | 183,491 (53%) | 916,907 (53%) | 154,712 (53%) | 755,739 (54%) | 28,779 (50%) | 161,168 (47%) | 141,426 (54%) | 679,260 (55%) | 42,065 (48%) | 237,647 (47%) |
| **Age** |  |  |  |  |  |  |  |  |  |  |
| 40-49 | 140,911 (41%) | 704,354 (41%) | 118,587 (41%) | 566,611 (41%) | 22,324 (39%) | 137,743 (40%) | 104,761 (40%) | 493,017 (40%) | 36,150 (41%) | 211,337 (42%) |
| 50-59 | 77,839 (22%) | 389,172 (22%) | 66,890 (23%) | 321,788 (23%) | 10,949 (19%) | 67,384 (20%) | 56,879 (22%) | 264,306 (22%) | 20,960 (24%) | 124,866 (25%) |
| 60-69 | 66,436 (19%) | 332,133 (19%) | 56,305 (19%) | 274,469 (20%) | 10,131 (18%) | 57,664 (17%) | 50,658 (20%) | 239,270 (20%) | 15,778 (18%) | 92,863 (18%) |
| 70-79 | 42,569 (12%) | 212,739 (12%) | 33,700 (12%) | 163,961 (12%) | 8,869 (15%) | 48,778 (14%) | 32,727 (13%) | 156,986 (13%) | 9,842 (11%) | 55,753 (11%) |
| 80-89 | 16,843 (4.9%) | 84,084 (4.8%) | 12,369 (4.3%) | 58,801 (4.2%) | 4,474 (7.8%) | 25,283 (7.4%) | 12,921 (5.0%) | 62,907 (5.1%) | 3,922 (4.5%) | 21,177 (4.2%) |
| 90-99 | 2,359 (0.7%) | 11,160 (0.6%) | 1,634 (0.6%) | 7,279 (0.5%) | 725 (1.3%) | 3,881 (1.1%) | 1,723 (0.7%) | 8,014 (0.7%) | 636 (0.7%) | 3,146 (0.6%) |
| 100+ | 24 (<0.1%) | 79 (<0.1%) | 18 (<0.1%) | 45 (<0.1%) | 6 (<0.1%) | 34 (<0.1%) | 13 (<0.1%) | 36 (<0.1%) | 11 (<0.1%) | 43 (<0.1%) |
| **Index of multiple deprivation quintile** |  |  |  |  |  |  |  |  |  |  |
| 1, least deprived | 77,178 (22%) | 398,819 (23%) | 62,346 (22%) | 311,255 (22%) | 14,832 (26%) | 87,564 (26%) | 55,817 (21%) | 273,825 (22%) | 21,361 (24%) | 124,994 (25%) |
| 2 | 75,431 (22%) | 380,816 (22%) | 61,846 (21%) | 301,956 (22%) | 13,585 (24%) | 78,860 (23%) | 55,372 (21%) | 264,317 (22%) | 20,059 (23%) | 116,499 (23%) |
| 3 | 67,893 (20%) | 336,145 (19%) | 56,532 (20%) | 269,880 (19%) | 11,361 (20%) | 66,265 (19%) | 50,702 (20%) | 236,316 (19%) | 17,191 (20%) | 99,829 (20%) |
| 4 | 64,838 (19%) | 318,431 (18%) | 55,462 (19%) | 261,603 (19%) | 9,376 (16%) | 56,828 (17%) | 49,817 (19%) | 230,361 (19%) | 15,021 (17%) | 88,070 (17%) |
| 5, most deprived | 61,514 (18%) | 298,627 (17%) | 53,254 (18%) | 248,005 (18%) | 8,260 (14%) | 50,622 (15%) | 47,908 (18%) | 219,391 (18%) | 13,606 (16%) | 79,236 (16%) |
| missing | 127 (<0.1%) | 883 (<0.1%) | 63 (<0.1%) | 255 (<0.1%) | 64 (0.1%) | 628 (0.2%) | 66 (<0.1%) | 326 (<0.1%) | 61 (<0.1%) | 557 (0.1%) |
| **Ethnicity** |  |  |  |  |  |  |  |  |  |  |
| White | 222,054 (64%) | 1,042,633 (60%) | 222,054 (77%) | 1,042,633 (75%) | 0 (0%) | 0 (0%) | 173,019 (67%) | 780,786 (64%) | 49,035 (56%) | 261,847 (51%) |
| South Asian | 9,913 (2.9%) | 53,615 (3.1%) | 9,913 (3.4%) | 53,615 (3.8%) | 0 (0%) | 0 (0%) | 8,752 (3.4%) | 46,192 (3.8%) | 1,161 (1.3%) | 7,423 (1.5%) |
| Black | 1,849 (0.5%) | 28,585 (1.6%) | 1,849 (0.6%) | 28,585 (2.1%) | 0 (0%) | 0 (0%) | 1,589 (0.6%) | 24,393 (2.0%) | 260 (0.3%) | 4,192 (0.8%) |
| Other/mixed | 49,993 (14%) | 236,587 (14%) | 49,993 (17%) | 236,587 (17%) | 0 (0%) | 0 (0%) | 42,108 (16%) | 194,071 (16%) | 7,885 (9.0%) | 42,516 (8.3%) |
| Missing | 57,478 (17%) | 340,767 (20%) | 0 (0%) | 0 (0%) | 57,478 (100%) | 340,767 (100%) | 30,454 (12%) | 160,147 (13%) | 27,024 (31%) | 180,620 (35%) |
| **Body mass index** |  |  |  |  |  |  |  |  |  |  |
| Not overweight (BMI<25 kg/m^2^) | 87,055 (25%) | 468,503 (27%) | 73,948 (26%) | 389,596 (28%) | 11,420 (20%) | 68,216 (20%) | 83,573 (32%) | 446,809 (36%) | 1,795 (2.1%) | 11,003 (2.2%) |
| Overweight (BMI>=25 kg/m^2^) | 187,230 (54%) | 829,241 (48%) | 159,849 (55%) | 698,099 (50%) | 20,610 (36%) | 100,367 (29%) | 176,109 (68%) | 777,727 (64%) | 4,350 (5.0%) | 20,739 (4.1%) |
| Missing | 72,696 (21%) | 435,977 (25%) | 55,706 (19%) | 305,259 (22%) | 25,448 (44%) | 172,184 (51%) | 0 (0%) | 0 (0%) | 81,154 (93%) | 477,443 (94%) |
| **Smoking** |  |  |  |  |  |  |  |  |  |  |
| Non-smoker | 131,126 (38%) | 770,020 (44%) | 110,412 (38%) | 634,276 (46%) | 17,379 (30%) | 113,565 (33%) | 110,594 (43%) | 639,739 (52%) | 17,197 (20%) | 108,102 (21%) |
| Current- or ex-smoker | 178,029 (51%) | 711,872 (41%) | 149,136 (52%) | 580,875 (42%) | 24,332 (42%) | 109,533 (32%) | 149,088 (57%) | 584,797 (48%) | 24,380 (28%) | 105,611 (21%) |
| Missing | 37,826 (11%) | 251,829 (15%) | 29,955 (10%) | 177,803 (13%) | 15,767 (27%) | 117,669 (35%) | 0 (0%) | 0 (0%) | 45,722 (52%) | 295,472 (58%) |
| **Harmful alcohol use** | 15,009 (4.3%) | 48,403 (2.8%) | 12,736 (4.4%) | 40,325 (2.9%) | 2,273 (4.0%) | 8,078 (2.4%) | 12,244 (4.7%) | 38,655 (3.2%) | 2,765 (3.2%) | 9,748 (1.9%) |
| **Chronic comorbidities** |  |  |  |  |  |  |  |  |  |  |
| Chronic liver disease | 3,079 (0.9%) | 9,388 (0.5%) | 2,665 (0.9%) | 7,985 (0.6%) | 414 (0.7%) | 1,403 (0.4%) | 2,677 (1.0%) | 8,026 (0.7%) | 402 (0.5%) | 1,362 (0.3%) |
| Chronic lung disease | 18,489 (5.3%) | 67,340 (3.9%) | 15,958 (5.5%) | 56,928 (4.1%) | 2,531 (4.4%) | 10,412 (3.1%) | 16,587 (6.4%) | 59,476 (4.9%) | 1,902 (2.2%) | 7,864 (1.5%) |
| Cardiovascular disease | 37,724 (11%) | 161,833 (9.3%) | 31,231 (11%) | 131,759 (9.5%) | 6,493 (11%) | 30,074 (8.8%) | 32,297 (12%) | 137,175 (11%) | 5,427 (6.2%) | 24,658 (4.8%) |
| Cerebrovascular disease | 11,088 (3.2%) | 48,257 (2.8%) | 9,113 (3.1%) | 38,897 (2.8%) | 1,975 (3.4%) | 9,360 (2.7%) | 9,315 (3.6%) | 40,145 (3.3%) | 1,773 (2.0%) | 8,112 (1.6%) |
| Diabetes mellitus | 30,189 (8.7%) | 125,437 (7.2%) | 26,796 (9.3%) | 110,240 (7.9%) | 3,393 (5.9%) | 15,197 (4.5%) | 28,416 (11%) | 117,694 (9.6%) | 1,773 (2.0%) | 7,743 (1.5%) |
| Depression | 84,747 (24%) | 338,515 (20%) | 74,089 (26%) | 291,291 (21%) | 10,658 (19%) | 47,224 (14%) | 72,634 (28%) | 284,641 (23%) | 12,113 (14%) | 53,874 (11%) |
| High cholesterol | 174,495 (50%) | 813,357 (47%) | 155,677 (54%) | 716,704 (51%) | 18,818 (33%) | 96,653 (28%) | 157,293 (61%) | 726,646 (59%) | 17,202 (20%) | 86,711 (17%) |
| Hypertension | 88,497 (26%) | 389,791 (22%) | 76,473 (26%) | 331,522 (24%) | 12,024 (21%) | 58,269 (17%) | 77,487 (30%) | 337,881 (28%) | 11,010 (13%) | 51,910 (10%) |
| Chronic kidney disease | 35,295 (10%) | 165,979 (9.6%) | 29,319 (10%) | 135,831 (9.8%) | 5,976 (10%) | 30,148 (8.8%) | 30,452 (12%) | 142,202 (12%) | 4,843 (5.5%) | 23,777 (4.7%) |
| **Median (IQR) consultations in year before cohort entry** | 12 (6, 22) | 7 (3, 15) | 13 (6, 23) | 8 (3, 16) | 9 (4, 17) | 4 (1, 11) | 14 (7, 24) | 10 (4, 18) | 6 (3, 13) | 3 (1, 8) |

## Table S3: Adjusted hazard ratios (95% CIs) comparing people with **severe psoriasis** matched to people without psoriasis*

| **Outcome** | **HR (95% CI)**  **(implicitly adjusted for age, sex, practice)** | **HR (95% CI)**  **(additionally adjusted for IMD, calendar period and comorbidities)** |
| --- | --- | --- |
| Dementia | 1.32 (1.25-1.39) | 1.23 (1.16-1.29) |
| Alzheimer’s dementia | 1.16 (1.07-1.26) | 1.11 (1.02-1.21) |
| Vascular dementia | 1.57 (1.44-1.72) | 1.45 (1.32-1.59) |

*Matched on first date of meeting our severe psoriasis definition rather than index date of main analysis (i.e., index date of main analysis was latest of: first record of a diagnostic morbidity code for psoriasis (recorded in primary or secondary care); practice registration plus one year; study start (1st April 1997), or 40th birthday).

## Table S4: Adjusted hazard ratios (95% CIs) for the association between psoriasis and dementia, in over 65s only, stratified by **frailty** **status**

|  | **Hazard ratio (95% confidence interval)*** | | |
| --- | --- | --- | --- |
|  | **Dementia** | **Alzheimer’s dementia** | **Vascular dementia** |
| **Not frail** | 1 (ref) | 1 (ref) | 1 (ref) |
| **Mild frailty** | 0.96 (0.92-1.01) | 1.05 (0.98-1.13) | 0.89 (0.82-0.97) |
| **Moderate frailty** | 0.85 (0.79-0.91) | 0.96 (0.86-1.08) | 0.88 (0.78-1.00) |
| **Severe frailty** | 1.02 (0.89-1.16) | 0.93 (0.74-1.17) | 1.13 (0.91-1.40) |

*Hazard ratios (95% CIs) implicitly adjusted for age, sex and practice, explicitly adjusted for deprivation (IMD), calendar period, and comorbidities, and allowing for interaction between psoriasis and frailty status.

## Table S5: Adjusted hazard ratios (95% CIs) for the association between psoriasis and dementia after additionally adjusting for time-updated **psoriatic arthritis status**

| **Outcome** | **HR (95% CI)* Without adjusting for psoriatic arthritis (for reference)** | **HR (95% CI)* Additionally adjusting for psoriatic arthritis** |
| --- | --- | --- |
| **Time-updated comorbidity-adjusted analysis (time-updated psoriatic arthritis status)** | | |
| Dementia | 1.02 (1.00-1.06) | 1.03 (1.00-1.06) |
| Alzheimer’s dementia | 1.02 (0.96-1.08) | 1.04 (0.98-1.10) |
| Vascular dementia | 1.09 (1.02-1.17) | 1.06 (1.00-1.13) |

*Hazard ratios (95% CIs) implicitly adjusted for age, sex and practice, explicitly adjusted for deprivation (IMD), calendar period, and comorbidities.
